# Tricuspid Valve Regurgitation: Current Understanding and Novel Treatment Options

**DOI:** 10.1016/j.jscai.2023.101041

**Published:** 2023-07-05

**Authors:** Alessandra Sala, Rebecca T. Hahn, Susheel K. Kodali, Michael J. Mack, Francesco Maisano

**Affiliations:** a Department of Cardiac Surgery, Vita-Salute San Raffaele University, IRCCS San Raffaele Hospital, Milan, Italy; b Department of Cardiology, Columbia University Medical Center/NewYork-Presbyterian Hospital, New York, New York; c Department of Cardiac Surgery, Baylor Scott & White Health, Plano, Texas

**Keywords:** outcomes, surgical treatment, timing, transcatheter interventions, tricuspid regurgitation, tricuspid valve disease

## Abstract

Managing patients with severe symptomatic tricuspid regurgitation (TR) remains extremely challenging, with a lack of consensus on when and how to treat it. Tricuspid valve pathology has been disregarded for a very long time because of the established belief that treating left-sided heart diseases would lead to the resolution or significant improvement of TR. Initially considered benign, severe TR has been found to be a strong predictor of prognosis. Despite the increasing prevalence and the disabling nature of this disease, the great majority of patients with clinically significant TR have seldom been considered for structural interventions. Numerous surgical and transcatheter treatment options are now available; however, optimal timing and procedural selection remain crucial aspects influencing outcomes. According to recent evidence in the literature, early referral is associated with good short and long-term outcomes, and various predictors of favorable outcomes following either surgical or transcatheter treatment have been identified. Evaluation by a multidisciplinary heart team with expertise in tricuspid valve disease is of paramount importance to identify adequate treatment for every patient.

## Highlights

• Management of severe symptomatic tricuspid regurgitation remains challenging.
• When and how to treat severe tricuspid regurgitation remains uncertain.
• Early referral is associated with good short- and long-term outcomes.
• Correct timing and adequate patient selection play the most important role.

## Central Illustration

**Figure:**
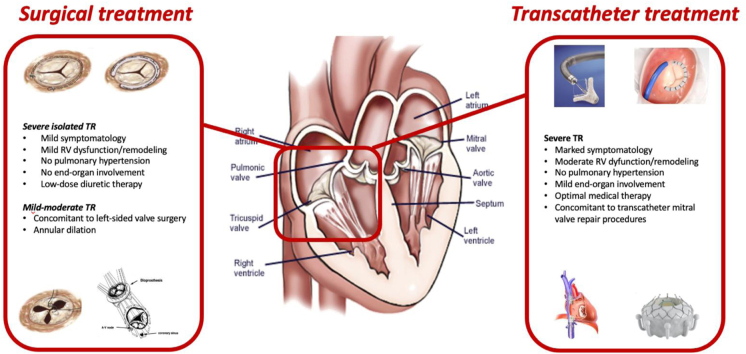

## Introduction

The incidence of moderate or severe tricuspid regurgitation (TR) increases with age; the prevalence in the general population reaches 1.5% and 5.6% in men and women, respectively.^1^ Treatment of TR has historically been ignored, given a belief that treatment of left-sided heart diseases would lead to resolution of secondary disease,^2^ and the acceptable mid-term outcomes of tricuspid valvectomy in the setting of intravenous drug use-associated endocarditis.^3^ Initially considered benign, severe TR has been found to be a strong predictor of prognosis in various disease states.^4^, ^5^, ^6^ Isolated TR of a least moderate-to-severe TR may be associated with a survival of only 72.0% at 1 year and 47.2% at 5 years.^7^ Despite its high prevalence and poor prognosis, most patients (>90%) are undertreated. This large unmet clinical need has caught the attention of both the cardiac surgery and cardiological community in recent years. However, managing patients with tricuspid valve (TV) disease remains controversial, and many uncertainties remain to date. Medical treatment remains the mainstay of therapy; however, in the absence of studies showing clinical benefit, diuretics and treatment of left heart disease or pulmonary hypertension remain class IIA recommendations.^8^ In addition to the lack of evidence, symptoms of TR are nonspecific and often attributed to concomitant disease processes accompanying secondary disease. Isolated TV surgery has been occasionally considered, but often too late in the course of the disease. As a consequence of the late referral, outcome data from the literature are discouraging, reporting in-hospital mortality rates as high as 10%.^9^^,^^10^ To overcome the limitations of surgical invasiveness, numerous devices for transcatheter treatment of TR have been developed. Nevertheless, a well-defined indication of “when” and “how” to treat patients with severe TR is still lacking. In fact, adequate patient selection and correct timing play the most important role in determining a good outcome following TR treatment, whether surgical or transcatheter.

In the present article, we aim to assess the natural history of TR, adequate patient stratification, and optimal timing for treatment. Furthermore, a review of the currently available surgical and transcatheter treatment strategies is provided.

## TV disease: diverse phenotypes, stages of the disease, and anatomical variants

Tricuspid regurgitation is defined as the inability of the valvular leaflets to adequately coapt during ventricular systole, reducing forward stroke volume despite increasing total right ventricle (RV) stroke volume. In the setting of increased RV volumes, ejection fraction (EF) may appear normal or increased. Although minimal or trivial TR may be considered a normal variant in structurally normal valves with no clinical impact, recent studies show that even mild or moderate TR is associated with a 29% increase in mortality compared to no TR.^11^

The TV is typically composed of 3 leaflets of unequal size, which by convention are named the anterior, posterior, and septal leaflets. Pathology studies have shown that there are variable numbers of leaflets; therefore, recently, a new nomenclature has been proposed in order to allow for better and clearer communication during the treatment of TV disease. Four major classes of leaflet morphologies have been identified: type I is the classic 3-leaflet morphology; type II is the 2-leaflet morphology because of the fusion of the anterior and posterior leaflets; type III is the 4-leaflet configuration with subcategories based on the location of the indentation/extra leaflet; and type IV has >4 leaflets.^12^ Studies have suggested that morphologic complexity may affect procedural outcomes for the transcatheter edge-to-edge repair (TEER) device.^13^^,^^14^

Current classification schemes for TR etiology have become more granular (Table 1), separating cardiac implantable electronic device (CIED) related TR from primary causes affecting the TV leaflets.^15^^,^^16^ In addition, secondary etiologies of TR are now separated into atrial secondary TR (ie, frequently with chronic atrial fibrillation and without concomitant systolic left heart disease and/or pulmonary vascular disease) and ventricular secondary TR (ie, associated with concomitant left heart and/or pulmonary vascular disease). In addition to differences in clinical characteristics, patients with atrial and ventricular secondary TR have several morphologic differences.^17^ Ventricular secondary TR is associated with RV dilatation, particularly in the mid-ventricle, resulting in tethering of the leaflets and, thus, malcoaptation with TR. Atrial secondary TR is associated with more marked right atrial and tricuspid annular dilatation, and only late in the disease process can RV dilatation and leaflet tethering be seen.

**Table 1:** New classification of tricuspid regurgitation etiology.

Causative disease process	Mechanism
Primary tricuspid regurgitation (5% to 10% of patients)	
Degenerative disease	Prolapse or flail leaflet
Congenital	Apical displacement of leaflet attachment (ie, Ebstein’s Anomaly or Cleft)
Acquired (ie, tumors, trauma, carcinoid, RHD, radiation)	Leaflet injury (ie, endocarditis, tumor, trauma, biopsy, and lead extraction) or infiltration/fibrosis (ie, carcinoid, rheumatic disease, and radiation valvulopathy)
Secondary tricuspid regurgitation^a^ (∼80% of patients)	
Ventricular secondary TR (V-STR)	
Left ventricular or valvular disease	Postcapillary PH (ie, HFrEF and left valve disease)
Pulmonary/pulmonary vascular disease	Precapillary PH (ie, chronic lung disease, CTEPH, and PAH)
Primary RV dysfunction/remodeling	RV dilation and dysfunction (ie, RV infarct and RV dysplasia)
Atrial secondary TR (A-STR)	
RA/TA dilatation	RA/TA dilatation (ie, related to age, atrial fibrillation, and HFpEF)
Lead-related TR (∼10% to 15%)	
Lead-related TR (LTR)	LTR-A (causative): leaflet impingement, perforation, valvular/subvalvular adhesions/restriction
	LTR-B (incidental)^a^: presence of CIED without interference valvular apparatus

CIED, cardiovascular implantable electronic device; CTEPH, chronic thromboembolic pulmonary hypertension; HFpEF, heart failure with preserved ejection fraction; HFrEF, heart failure with reduced ejection fraction; LTR, lead-related tricuspid regurgitation; PAH, pulmonary arterial hypertension; PH, pulmonary hypertension; RA, right atrial; RV, right ventricular; TA, tricuspid annular; RHD, right heart disease; A-STR, atrial secondary TR; LTR, lead related TR; V-STR, ventricular secondary TR.

a CIED may be present and, although not the causative mechanism of tricuspid regurgitation, may impact transcatheter device choice.

## Primary TR

Primary TR results from anatomic abnormalities intrinsically affecting the TV or the subvalvular apparatus and accounts for a minority of cases of significant TR, reported as between 5% and 10% of patients.^7^^,^^18^ Etiologies include congenital anomalies, including Ebstein’s anomaly or cleft valve associated with atrioventricular canal defect. Acquired diseases of the TV include degenerative disease (ie, prolapse), rheumatic disease, infective endocarditis, carcinoid heart disease, tumors, trauma, and iatrogenic causes (ie, biopsy). A single-site study from a tertiary care center suggests the main primary etiologies are endocarditis in 222 (47.2%), degenerative or prolapse in 86 (18.3%), and prosthetic valve failure in 79 (16.8%).^7^ TV endocarditis is associated with worse outcomes compared to degenerative disease.^7^

## Secondary (functional) TR

Secondary TR is more common than primary TR, seen in >90% of cases in contemporary series.^7^^,^^18^ Because of the interdependence of the left ventricle (LV) and RV and the RV remodeling that occurs in response to changes in pulmonary arterial and/or venous pressures (or compliance), ventricular secondary TR is most frequently seen in the setting of left heart disease (either myocardial or valvular) and pulmonary hypertension (both precapillary and postcapillary).^19^ Recent single-site studies suggest the main secondary etiologies, including left heart disease in 4664 (54.4%), atrial function in 2086 (24.3%), and pulmonary disease in 1454 (17.0%).^7^ Severe TR is often seen in 23% to 37% of patients after mitral valve replacement, in most cases being diagnosed as late as 10 years following the procedure.^20^^,^^21^ Outcomes with each of these etiologies vary, with TR in the setting of pulmonary arterial hypertension having the worst outcomes.^7^^,^^22^^,^^23^ Atrial secondary TR is more common in females and may be associated with atrial fibrillation and heart failure with preserved EF.^24^^,^^25^ Multiple studies have suggested that patients with ventricular secondary TR have worse outcomes than atrial secondary TR^26^^,^^27^; however, other studies suggest that there is no statistically significant difference in outcomes for atrial versus ventricular secondary TR on multivariable analysis.^7^ Risk scores have been developed to assess both natural history outcomes^7^ and operative mortality.^28^, ^29^, ^30^ However, all scores have limitations, and these risk scores should be integrated with other clinical factors when managing individual patients.^16^

## Cardiac implantable electronic devices

The development of relevant TR in patients undergoing de novo implantation of CIEDs is reported to be as high as 39%^31^ and is a predictor of TR progression.^32^ The overall prevalence of CIED-related TR is difficult to ascertain, ranging from an overall prevalence of 0.5% to 5%.^7^^,^^18^ Current trials of transcatheter devices have enrolled up to 35% of patients with CIED.^33^ The location of the CIED has been associated with the development of TR.^34^^,^^35^ Damage to the TV leaflets rarely occurs (<3%)^36^; however, improvement in TR with CIED lead removal has been reported in only 35% of patients.^35^ Although patients with CIED-related TR have worse long-term survival, the improvement in valve function following lead extraction contributes to a significant reduction in mortality.^35^

## Management of patients with TR

### Guideline-directed medical management of TR

Early in the disease process, in response to TR and central venous congestion, RV compensatory mechanisms and remodeling contribute to maintaining adequate hemodynamic compensation and cardiac output. Patients tend to be asymptomatic with no medical therapy. However, compensatory mechanisms are limited in the RV compared to the LV.^37^ As the disease progresses, patients develop pulmonary and central venous congestion, with the occurrence of exertional dyspnea, orthopnea, and peripheral edema. Long-lasting TR further leads to a disproportionate RV dilation along the free wall, resulting in a more spherical RV shape and progressive RV dysfunction. This aggravates TR and results in multiorgan involvement, with the development of hepato-splenomegaly, reduced renal function, pleural effusions, ascites, and initial right heart failure (RHF) episodes. In the late stages of the disease, overt chronic RHF develops, with end-organ damage (hepatorenal and gastrointestinal dysfunction) because of chronic RV volume overload, with severe RV dysfunction and remodeling^38^; this reflects the role of hepatorenal scores to predict outcomes in TR.^30^^,^^39^ Patients experience frequent RHF hospitalizations, despite optimal medical management, and symptoms are mostly related to low cardiac output, such as fatigue, asthenia and poor functional capacity.^40^

Therefore, untreated TR leads to the development of a vicious cycle of increasing RV dilation and increasing degrees of regurgitation. Although initial RV remodeling can be accompanied by normal RV function, long-standing TR results in maladaptive RV remodeling, with changes in RV geometry that cause papillary muscle displacement, abnormal leaflet tethering, large coaptation gaps, which ultimately lead to torrential TR and severe RV dysfunction.^41^^,^^42^ This cycle should be interrupted early on in order to positively impact on long-term prognosis of patients affected by TR.

## Risk assessment and indications for intervention

### Right heart assessment and risk stratification of patients

Right ventricle physiology and function are less studied and less understood than the LV. Initially considered a passive conduit with minimal pumping capability, it is now well demonstrated that RV systolic function and diastolic load are extremely important to cardiac output.^43^ However, RV function is dependent not only on intrinsic contractile function but also on loading conditions; thus, to better stratify patients with severe TR, a comprehensive assessment of RV size and function should be performed in a euvolemic state.

Noninvasive assessment of the RV is a complex task, requiring the integrated evaluation of multiple parameters, and should take advantage of emerging imaging modalities, such as speckle-tracking, 3-dimensional (3D) echocardiography, cardiac computed tomography, and cardiac magnetic resonance (Table 2).^15^^,^^44^^,^^45^ RV dilation and systolic function are extremely relevant parameters in evaluating and managing patients with significant TR because of their prognostic relevance. Patients with RV systolic dysfunction measured by longitudinal function such as tricuspid annular plane systolic excursion (TAPSE)^46^ or RV free-wall strain^47^^,^^48^ experience increased mortality when untreated, and increased morbidity and mortality are seen following isolated TV surgery.^49^

**Table 2:** Right heart assessment.

Imaging modality	Applications
TTE/TEE	Evaluation of RV function - TAPSE, s’TDI, FAC (2D methods) - RVEF%, stroke volume (3D methods) - RV global and free-wall longitudinal strain - TAPSE/sPAP ratio Diagnosis of pulmonary hypertension - sPAP Contractile reserve by stress imaging - Increase of TAPSE
CCT	RV volumetric measures
CMR	Evaluation of RV function - Volumetric measures (RV-EDV) - Strain analysis Assessment myocardial fibrosis - Tissue remodeling (LGE and extracellular volume) Contractile reserve by stress imaging
RHC	Diagnosis of pulmonary hypertension - sPAP, mPAP, and dPAP - Pulmonary vascular resistance Evaluation of RV function - Transpulmonary gradient - RV stroke work

CCT, cardiac computed tomography; CMR, cardiac magnetic resonance; dPAP, diastolic pulmonary artery pressure; FAC, fractional area change; LGE, late gadolinium enhancement; mPAP, mean pulmonary artery pressure; RHC, right heart catheterization; RV, right ventricle; RV-EDV, right ventricular end diastolic volume; RVEF, right ventricular ejection fraction; s’TDI, systolic tissue doppler imaging; sPAP, systolic pulmonary artery pressure; TAPSE, tricuspid annular plane systolic excursion; TEE, transesophageal echocardiography; TTE, transthoracic echocardiography; 2D, 2-dimensional; 3D, 3-dimensional.

In patients undergoing transcatheter TV interventions, the presence of low TAPSE^50^ and reduced global function measured by 3D-RVEF^51^^,^^52^ is associated with adverse outcomes. More specifically, patients with mid-range RV function (TAPSE 13-17 mm)^50^ and patients with a 3D-RVEF >45%^51^^,^^52^ experienced survival benefits and the greatest clinical improvement. RV function, however, is dependent on both preload and afterload. The high preload condition associated with TR results in larger RV stroke volumes which increases current measures of RV function, masking the presence of reduced contractile function. On the other hand, RV contractility may increase in the setting of increasing afterload, a metric known as RV-pulmonary artery (PA) coupling. When the RV function fails to compensate for the increase in afterload, there is RV-PA uncoupling. Indexing RV function to afterload has been proposed as a better measure of RV function for specific loading conditions. Noninvasively derived RV-PA coupling is gained through TAPSE/systolic PA pressure ratios, and higher ratios have been shown to be associated with lower all-cause mortality and fewer hospitalizations for RHF.^53^ However, recent data have shown that the diagnostic sensitivity of echocardiography in accurately measuring systolic PA pressure is significantly reduced by the presence of various clinical factors, including greater severity of TR and worse RV and LV function.^54^ Right heart catheterization remains, therefore, the gold standard for the invasive assessment of the right heart physiology, providing information regarding the severity and mechanism of pulmonary hypertension, pulmonary vascular resistance, preload conditions, and RV function.^55^ Mean PA pressure, diastolic PA pressure, transpulmonary gradient, pulmonary vascular resistance, and RV stroke work have all been identified as hemodynamic predictors of worse outcomes (1-year mortality, heart failure hospitalizations, and reintervention) in patients undergoing transcatheter TV repair (TVr) for severe TR.^23^

In patients with pulmonary arterial hypertension or chronic thromboembolic pulmonary hypertension, several exercise variables during cardiopulmonary exercise testing have proven to be useful in establishing the severity of functional impairment, predicting prognosis, and assessing the efficacy of interventions.^55^^,^^56^ The utility of stress testing for patients with TR has not been studied. Finally, the detection of myocardial fibrosis by cardiac magnetic resonance or by speckle-tracking echocardiography has recently demonstrated prognostic relevance in RV failure and might represent a promising tool to define the optimal timing of intervention in severe TR.^57^

### Operative risk scores

Besides assessing right heart physiology and hemodynamic status, many other parameters have been identified to improve patients’ stratification (Table 3). Two dedicated risk scores have been proposed to predict the in-hospital outcome of patients following isolated TV surgery.^28^^,^^29^ The Society of Thoracic Surgeons TV score included age, sex, stroke, hemodialysis, EF, lung disease, New York Heart Association (NYHA) class, reoperation, and urgent or emergency status. A simple risk score from 0 to 10+ was associated (*P* < .001) with incremental increases in predicted mortality and major morbidity.^28^ The TRI-SCORE is based on 8 parameters: age, NYHA functional class, RHF signs, a daily dose of furosemide, renal insufficiency, elevated total bilirubin, LFEF, and moderate/severe RV dysfunction. These risk scores have not been validated in patients undergoing transcatheter interventions.

**Table 3:** Patient stratification parameters.

	Parameters
Right ventricle	• Moderate/severe RV dysfunction (TAPSE <17 mm and s’TDI <9.5 cm/s) • TAPSE <17 mm • 3D-RVEF <45% • RV-PA coupling (TAPSE/sPAP ratio <0.406)
Cardiopulmonary hemodynamic profile	• sPAP ≥50 mm Hg • mPAP >30 mm Hg • dPAP >20 mm Hg • TPG >17 mm Hg • PVR >5 WU
Clinical	• Age ≥70 years • NYHA class III-IV • Daily dose of furosemide ≥125 mg • RHF signs (ascites, peripheral edema, and severe jugular venous distension)
End-organ	• Glomerular filtration rate <30 ml/min • Elevated total bilirubin
Left ventricle	• LV-EF <60%

dPAP, diastolic pulmonary artery pressure; LV-EF, left ventricle ejection fraction; mPAP, mean pulmonary artery pressure; NYHA, New York Heart Association; PVR, pulmonary vascular resistance; RHF, right heart failure; RV, right ventricle; RV-PA, right ventricle-pulmonary artery; s’TDI, systolic tissue doppler imaging; sPAP, systolic pulmonary artery pressure; TAPSE, tricuspid annular plane systolic excursion; TPG, transpulmonary gradient; 3D-RVEF, 3-dimensional right ventricular ejection fraction.

A new clinical and functional classification has recently been proposed (Table 4) based not only on TR grade and RV remodeling/function but also on RHF episodes, symptoms, end-organ involvement, and medical therapy.^58^ Patients are divided into 5 stages according to disease progression, ranging from asymptomatic with moderate TR (Stage 1) to overt RHF patients, regardless of optimal medical therapy, with torrential TR (Stage 5). These theoretical stages lack specific cut-off values capable of guiding the decision-making process of optimal patient management and timing but introduce the concept of early and late presentation of the disease.

**Table 4:** Clinical and functional classification of patients with tricuspid regurgitation.

Stage	Characteristics
Stage 1	• TR less than moderate • No symptoms • Normal RV function and remodeling • Normal TV annulus, leaflet coaptation • No tethering • No medical treatment
Stage 2	• TR greater than moderate • No symptoms • Normal RV function, mild remodeling • Mild annular dilation, mildly abnormal coaptation • Mild tethering (<8 mm) • No treatment or low-dose diuretics
Stage 3	• Severe TR • Vague symptoms • Mild RV dysfunction and remodeling • Annular remodeling, abnormal leaflet coaptation • Abnormal tethering (<8 mm) • Diuretic treatment
Stage 4	• Severe TR • Current or previous episodes of RHF • Greater than moderate RV dysfunction and remodeling • Moderate-severe annular remodeling, coaptation gap • Abnormal with varying degrees of tethering • Moderate-high doses of diuretics
Stage 5	• Torrential TR • Overt RHF and/or end-organ damage • Severe RV dysfunction and remodeling • Severe annular remodeling, large coaptation gap • Severe tethering (>8 mm) • Multiple admissions for RHF, IV diuretics, or high-dose combination diuretic therapy

IV, intravenous; RHF, right heart failure; RV, right ventricle; TR, tricuspid regurgitation; TV, tricuspid valve.

## Guideline indications for intervention

Most recommendations of the current European^44^ and American^8^ guidelines for managing valvular heart disease involve patients undergoing concomitant left-sided valve operations (Table 5). TV surgery should be performed in patients with severe TR (class I) or mild-to-moderate TR with annular dilation (≥40 mm or >21 mm/m^2^ by bidimensional transthoracic echocardiography) or prior evidence of RHF (American College of Cardiology/American Heart Association guidelines) in patients undergoing left-sided valve surgery (class IIa).

**Table 5:** Current European and American guidelines for the treatment of tricuspid regurgitation.

2021 ESC/EACTS guidelines	2020 ACC/AHA guidelines
Class I 1. Severe TR (primary or secondary) undergoing left-sided valve surgery (B-C) 2. Severe symptomatic primary TR without severe RV dysfunction I	Class I 1. Severe TR undergoing left-sided valve surgery (B)
Class IIa 1. Moderate primary TR or mild or moderate secondary TR with dilated TA (>40 mm) undergoing left-sided valve surgery (B-C) 2. Asymptomatic severe primary TR with RV dilation, appropriate for surgery I 3. Severe secondary TR (with or without previous left-sided surgery), with symptoms or RV dilation, in the absence of severe LV of RV dysfunction and PH (B)	Class IIa 1. Progressive TR undergoing left-sided valve surgery in case of either TA dilation (>40 mm) or prior signs/symptoms of RHF (B) 2. Signs/symptoms of RHF and severe primary TR (B) 3. Signs/symptoms of RHF and severe isolated secondary TR because of TA dilation without PH, poorly responsive to medical therapy (B)
Class IIb 1. Transcatheter treatment of symptomatic secondary TR in inoperable patients at a Heart Valve Center with expertise I	Class IIb 1. Asymptomatic with severe primary TR and progressive RV dilation or systolic dysfunction I 2. Signs/symptoms of RHF and severe TR following left-sided valve surgery, in the absence of PH or severe RV dysfunction (B)

ACC, American College of Cardiology; AHA, American Heart Association; B, systematic reviews, individual cohort studies; C, evidence from case series and expert opinion; EACTS, European Association for Cardio-Thoracic Surgery; ESC, European Society of Cardiology; LV, left ventricle; PH, pulmonary hypertension; RHF, right heart failure; RV, right ventricle; TA, tricuspid annulus; TR, tricuspid regurgitation.

For patients with isolated tricuspid surgery, the American Heart Association guidelines are more conservative and suggest waiting for the development of signs or symptoms of RHF before recommending TV surgery in the absence of pulmonary hypertension and in patients who are poorly responsive to medical therapy (class IIa). Only a class IIb recommendation is given in asymptomatic patients with primary severe isolated TR and progressive RV dilation/dysfunction and in patients with signs and symptoms of RHF and severe TR who have undergone previous left-sided valve surgery, in the absence of severe pulmonary hypertension or severe RV dysfunction. However, the development of persisting symptoms usually occurs only in the advanced stages of the disease, being the clinical manifestation of RV failure with organ damage. In contrast, the European Society of Cardiology/European Association for Cardio-Thoracic Surgery guidelines^44^ strongly support an earlier surgical referral to achieve low in-hospital mortality and better postoperative outcomes. Surgery is indicated in patients with severe symptomatic primary TR without severe RV dysfunction (class I) and should be considered in asymptomatic or mildly symptomatic patients with severe primary or secondary TR with RV dilation in the absence of severe LV or RV dysfunction and pulmonary hypertension (class IIa). Furthermore, for the first time, European guidelines include the option for transcatheter treatment of symptomatic secondary TR in inoperable patients when referred to a Heart Valve Center with specific expertise (class IIb).

The timing of intervention for patients with TR remains controversial: ideally, it should be carried out sufficiently early to avoid irreversible organ failure and RV dysfunction.^49^ However, both for surgical and transcatheter TV intervention (TTVI), correct timing is crucial to avoid futility in patients with end-stage heart failure or end-organ damage.

## Surgical and transcatheter interventions

### Isolated TV surgery

Even though surgery is the gold standard treatment for severe TR, it is rarely performed. The majority of TV operations are performed concomitantly to left-sided valve surgeries, whereas only approximately 14% are performed in isolation.^9^^,^^59^^,^^60^ This occurs mainly in response to the historically reported high in-hospital mortality rates following TV surgery and the poor long-term outcomes. Older studies have, in fact, reported an in-hospital mortality ranging from 8.8% to 37% and a 55% mortality at 5 years.^9^^,^^61^, ^62^, ^63^ However, the baseline clinical presentation of such patients and the stage of the disease may have negatively impacted the outcome. Factors associated with disease duration and late clinical presentation, such as NYHA functional class III/IV, moderate and severe RV dysfunction, decompensated heart failure, and advanced end-organ liver disease, have been found to be independent predictors associated with in-hospital mortality.^10^^,^^64^ Therefore, patients referred to TV correction late in the disease course experience high morbidity and mortality after surgery, further supporting the idea that TV surgery is a high-risk procedure and further delays or even rejects the referral for surgery.

More recent studies have shown that surgical treatment of TR in the early stages of the disease, without prominent symptomatology, RV dilation or dysfunction, and without organ involvement, more frequently leads to TVr with low in-hospital mortality, fewer postoperative complications, and shorter postoperative length-of-stay whereas, patients treated in more advanced stages experience higher in-hospital mortality (15.3%), postoperative complications (such as acute kidney injury and low cardiac output syndrome), longer intensive care unit and hospital lengths-of-stay.^65^^,^^66^ Moreover, early-stage patients experience 100% survival at 5 years with no further hospitalizations for RHF compared to late-stage patients whose survival is approximately 60% and 1 out of 5 patients experience at least 1 hospitalization for RHF.^67^

## Surgical repair versus replacement

Tricuspid valve repair remains the preferred technique in patients requiring surgery.^8^^,^^44^ Surgical TVr mainly focuses on tricuspid annuloplasty, which aims to reduce annulus diameter and cross-sectional valve area and restore normal 3D valve anatomy. Annuloplasty can be performed with suturing techniques or with the implantation of rings. The current gold standard for surgical repair is ring annuloplasty with an incomplete semi-rigid prosthetic ring. Ring annuloplasty, as compared to suture techniques (such as Kay bicuspidalization or De Vega suture annuloplasties), is generally associated with improved long-term survival and event-free survival and correlates with a trend toward fewer TV reoperations.^62^^,^^68^^,^^69^ In addition, multiple clinical, anatomic, and surgical tricuspid annular repair failure predictors have been identified, including leaflet tethering associated with ventricular secondary TR.^70^ Surgical techniques that address leaflets include the clover technique or leaflet augmentation, with reported promising results, especially in complex lesions and significant leaflet tethering scenarios.^71^, ^72^, ^73^

Overall, surgical TV replacement (TVR) is performed in a minority of patients, approximately 10% to 15% of reported cases. Hesitance is mainly related to the very high immediate perioperative morbidity and mortality rates, reported as high as 10.9%, which has not changed significantly during the last decade.^9^^,^^74^ Also, studies have indicated that TVr is more beneficial than TVR regarding all-cause mortality.^75^^,^^76^ Moreover, approximately one-third of patients undergoing TVR receive a permanent pacemaker prior to discharge, which remains a major concern.^77^ Generally, patients are more likely to undergo valve replacement in case of primary/organic TR with extensive leaflet pathologies such as infective endocarditis, rheumatic disease, or iatrogenic causes) or in secondary TR when the TV leaflets are excessively tethered, or the annulus is severely dilated.^78^ In fact, a tethering height >0.51 cm and a tethering area >0.80 cm^2^ were predictive of moderate-to-severe TR at 1 year following TV annuloplasty.^79^ Valve replacement is also associated with improved survival in patients with tricuspid annular diameter >44 mm as compared to valve repair.^80^

The optimal prosthesis for TVR is still controversial. However, a recent meta-analysis suggested an equal risk of in-hospital and late mortality, reoperation rate, and 5-year valve failure in patients undergoing TVR with a mechanical versus biological prosthesis.^81^ As there appears to be no superiority of one prosthesis over the other, the possibility of performing new percutaneous procedures of TV-in-valve implantation following bioprosthetic failure contributes to tipping the scale in favor of tissue valves.

## Transcatheter TV interventions

Transcatheter TV interventions present several technical challenges, mainly because of the complexity of the tricuspid anatomy (large safety and efficacy of these devices are expanding. Retrospective studies have reported a reduction annulus, thin valvular leaflets, and variable anatomy) and difficult visualization leading to challenging intraprocedural guidance. The spectrum of TTVI includes leaflet coaptation devices, annular repair devices, heterotopic caval valve implantation, and transcatheter TV replacement (TTVR) with orthotopic valve implantation (Table 6). Evidence of in TR grade, symptomatic improvement (reduced RHF hospitalizations, 26 ± 3% vs 47 ± 3%), and lower mortality at 1 year in patients treated with various devices compared to medical treatment alone.^82^, ^83^, ^84^ Moreover, reverse remodeling of the RV, improved cardiac output, and reduced liver enzymes were also reported.^85^, ^86^, ^87^ Despite these encouraging results, confirmation of these findings in randomized controlled trials is needed. More importantly, the indication and timing of TTVI are of paramount importance and should take into account patients’ clinical characteristics, disease stage, end-organ function, and anatomical factors (Central Illustration). In fact, patients treated in the late stages of the disease, with pronounced RV dysfunction (defined by low RVEF and TAPSE <13 mm), may not benefit from the reduction in venous congestion and reverse remodeling, impacting clinical events.^51^^,^^52^^,^^50^ Furthermore, NYHA class IV, pulmonary hypertension, renal dysfunction, and significant hepatic congestion were all independent predictors of all-cause mortality, with estimated 1-year mortality approaching 50%.^53^^,^^54^^,^^88^

**Table 6:** Transcatheter treatment options and early results.

Device	Procedural success	30-d outcomes
Leaflet approximation		
TriClip (TRILUMINATE study)	100%	TR less than or equal to moderate: 71% NYHA class I-II: 83% MAE and all-cause mortality: 7.1%
PASCAL (CLASP study)	100%	TR less than or equal to moderate: 52% NYHA I-II: 89% MAE and all-cause mortality: 5.9%
Annuloplasty		
Cardioband (TRI-REPAIR and TriBAND studies)	93%	TR less than or equal to moderate: 59% NYHA class I-II: 74% MAE and all-cause mortality: 8.2%
Heterotopic valve implantation		
TricValve (TRICUS-EURO study)	94%	NYHA class I-II: 79.4% All-cause mortality: 8.5% HF hospitalization: 20%
Tricento (Multicenter registry)	100%	NYHA calss I-II: 65% MAE and all-cause mortality: 24%
Orthotopic valve implantation		
GATE bioprosthesis (Multinational experience)	87%	TR less than or equal to moderate: 100% NYHA class I-II: 62% MAE and all-cause mortality: 10%
LuX-Valve (TRAVEL study)	97.8%	TR less than or equal to moderate: 100% NYHA class I-II: 100% MAE and all-cause mortality: 17%
EVOQUE bioprosthesis (TRISCEND study)	92%	TR less than or equal to moderate: 100% NYHA class I-II: 76% MAE and all-cause mortality: 3.8%

HF, heart failure; MAE, major adverse events; NYHA, New York Heart Association; TR, tricuspid regurgitation.

**Central Illustration:**
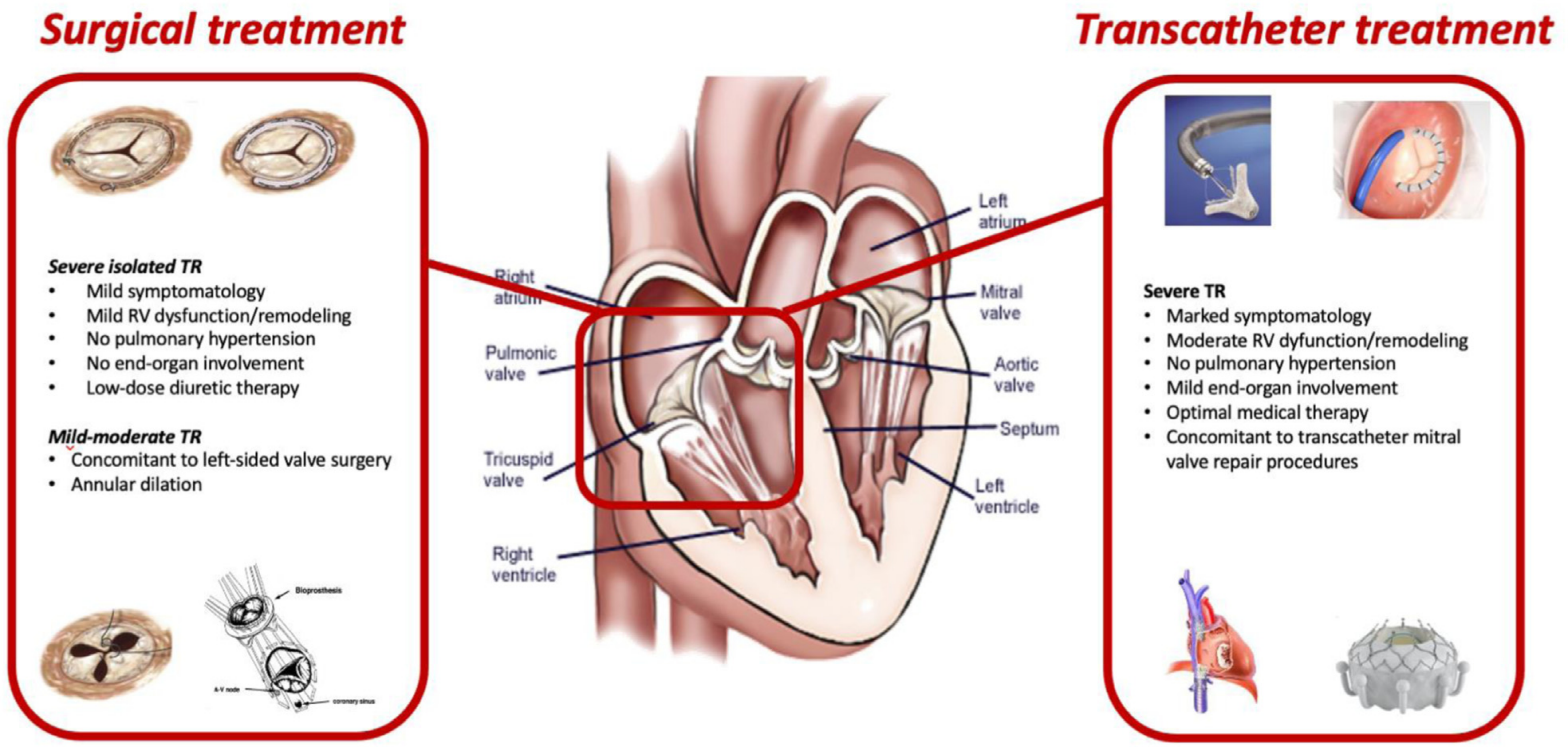
The available surgical and transcatheter treatment options for severe tricuspid regurgitation. RV, right ventricle; TR, tricuspid regurgitation.

### Tricuspid TEER repair

At present, the most widely applied technique is tricuspid TEER repair (T-TEER).^89^^,^^90^ T-TEER is performed under general anesthesia and with transesophageal echocardiographic guidance by an interventional imager.^91^, ^92^, ^93^ Compared to mitral TEER, T-TEER is more complex for several reasons, 2 above all: the complexity of TV anatomy known to have multiple leaflets and coaptation lines,^12^ and the marked challenge of transesophageal echocardiographic imaging of a far field, thin-leaflet TV.^93^^,^^94^ Nevertheless, results are constantly and rapidly improving because of the advanced knowledge and experience of both the proceduralist and the interventional imager and the introduction of new imaging software and devices.^93^

The newest generation devices include 2 main platforms: the TriClip G4 (Abbott Vascular) and the PASCAL (Edwards Lifesciences). Prior to the introduction of the dedicated TV delivery system, T-TEER was performed by adapting the mitral delivery system with an off-label approach in compassionate cases. This approach was limited by 2 main challenges: the device was unsupported by the interatrial septum and had the tendency to fall into the RV with limited height for proper leaflet grasping, and the trajectory was rarely coaxial because of the angle between the vena cava and the tricuspid annular plane. These challenges have been solved by developing a new dedicated TriClip delivery system. In addition, the latest generation clip technology has introduced 2 major advances, instrumental for tricuspid interventions: longer clip arms to reach the leaflets in patients with wider gaps^95^ and independent gripper activation to enable independent grasping. The PASCAL device has a nitinol frame and works according to a similar concept of clasping leaflets to individual paddles and approximating leaflet edges by the closure of the device. In addition to the clasping function, the PASCAL device has a spacer incorporated in the design to fill the coaptation gap. Because the frame is nitinol and the paddles are wide with frictional elements positioned across the top of the paddles (instead of longitudinally down the length of the clip arm in the TriClip device), it potentially reduces leaflet stress and trauma. In addition, the PASCAL device can be easily retrieved by elongating the nitinol structure, reducing the risk of chordal entanglement.

Results from retrospective studies assessing leaflet approximation devices have shown a durable reduction to moderate-or-less TR in approximately 70% of patients, together with symptomatic improvement (40% reduction in rehospitalization rates) and lower mortality at 1 year.^82^^,^^89^^,^^96^^,^^97^ Anatomical limitations, such as large coaptation gap (>7-10 mm) and non-anteroseptal location of the TR jet, pacemakers causing the TR, and dense chordal structures, have, however, been identified as predictors of procedural failure.^16^ The TRILUMINATE trial has shown that a reduction of at least 1 degree of TR is associated with improved symptoms at follow-up.^89^^,^^98^ However, residual TR is associated with worse outcomes.^14^^,^^99^

According to the latest postmarket approval data, outcomes have improved significantly using the TriClip G4 platform. The bRIGHT registry recently reported 1-year results (presented at PCR London Valve, November 2022) of 151 patients, with TR reduced to less than or equal to moderate in 86% of patients, NYHA class I/II in 77% of patients (compared to baseline of 21%) with a 21-point improvement in Kansas City Cardiomyopathy Questionaire-OS. The composite outcome of mortality or TV reintervention/reoperation at 1 year was 17.6%, and the estimated reduction in heart failure hospitalizations was 44%. This trial confirmed that TR less than or equal to moderate (compared to TR less than or equal to severe) was associated with a significant reduction in mortality (7.9% vs 26.1%, *P* = .0015). In addition, the recently released results of the TRILUMINATE Pivotal study, comparing T-TEER versus medical therapy alone, showed that T-TEER is safe (98.3%), with a favorable composite outcome of death from any cause, TV surgery, heart failure hospitalizations, and improvement in quality-of-life driven by the Kansas City Cardiomyopathy Questionaire improvement. Analysis of individual outcomes showed no difference between device and medical therapy in either mortality or heart failure hospitalizations despite a significant reduction in the severity of TR (at 30 days, 87% of patients versus 4.8% had no more than moderate TR).^100^ Further analysis of these results as well as the results of ongoing T-TEER randomized controlled trials CLASP II TR and TRACE-NL) may help define the appropriate patient population for treatment and the expected benefits of therapy.

Other randomized pivotal trials comparing guideline-directed medical therapy alone to T-TEER in addition to medical therapy are ongoing to address whether device therapy can improve mortality and heart failure hospitalizations (CLASP II TR and TRI-FR trials).

### Annular repair devices

To overcome the limitation of T-TEER, various transcatheter techniques have been developed and applied, including annuloplasty. The Cardioband system (Edwards Lifesciences) implantation is performed in selected centers worldwide. However, penetration of the procedure has been slow because of the high demand for procedural imaging. The Cardioband is implanted under echocardiographic and fluoroscopic guidance by inserting multiple anchors on the annulus between the leaflets and the right coronary artery. Results following Cardioband implantation (TRI-REPAIR and TriBAND studies) reported a reduction in TR grade to moderate-or-less in more than 70% of cases, both at 6 months and 2 years follow-up, associated with symptomatic improvement (NYHA class I/II) in 80% of patients.^101^^,^^102^ Right coronary artery adverse events have been reported frequently, although the risk of the procedure has been low. Furthermore, by addressing the annulus and maintaining the native tricuspid leaflets, the Cardioband tricuspid system preserves the option for further interventions, such as leaflet repair or valve replacement.

Other annuloplasty techniques are in development or in early human use.^103^ Current limitations of annular devices include excessive annular dilation, large coaptation gaps, and short or retracted septal leaflets.^16^

### Valve replacement devices

Multiple TTVR devices are under clinical and preclinical investigation and can be divided into 2 categories: heterotopic and orthotopic TVR. The former is characterized by deploying a valve (or valves) in 1 or both venae cavae to reduce the backflow in the venous system and relieve venous congestion and TR-related symptoms. Dedicated caval valve implantation devices have been developed, such as the TricValve (TricValve, P&F Products Features Vertriebs) and the Tricento (NVT, Hechingen, Germany). Although the TricValve features 2 valves implanted separately in the superior and inferior vena cava,^104^ the Tricento consists of a custom-made single valved stent linking both venae cavae.^105^ Successful implantations of both devices have been reported. These approaches are palliative and provide symptom alleviation in patients severely symptomatic despite optimal medical therapy and considered inoperable. However, the long-term implications of such therapies need to be evaluated.

Orthotopic valves are implanted in the native TV position. Valve performance and absence of residual TR seem superior in patients undergoing orthotopic TTVR with devices such as the Gate valve (NaviGate Cardiac Structures Inc),^106^ the EVOQUE TVR system (Edwards Lifesciences LLC),^33^^,^^107^ the Lux-Valve (Jenscare Biotechnology)^108^^,^^109^ and Cardiovalve (Cardiovalve Ltd).^110^ Early devices were implanted via the transatrial surgical route,^106^^,^^108^ and the transfemoral venous approach is now preferred. Preliminary results of the TRISCEND feasibility study regarding the EVOQUE bioprosthesis have been encouraging, with low 30-day all-cause mortality (3.6%), cardiovascular mortality (1.8%), with significant improvement in symptoms, function, and quality of life, but high rates of major adverse events (primarily bleeding in 27%), and new pacemaker requirement (11%).^33^ In addition, 98% of patients had a reduction in TR to none/trace or mild. In the setting of the near complete elimination of TR, the increase in effective afterload has raised concerns over acute RV failure; however, this has not been reported in recent series.^33^^,^^107^ Early in-human experience with transfemoral implantation of the Intrepid valve (Medtronic) and the Topaz valve (TRiCares)^111^ have been reported in individual patients.

A recent meta-analysis of available trials and registries suggests that for all evaluated devices, there is a significant reduction in TR with a reduction in RV dimensions and functions.^112^ However, despite this reduction in overall function, there is an increase in forward stroke volume and associated improvements in function and quality of life, with the predicted mortality at 30 days, 6 months, and 1 year of 5%, 10%, and 25%, respectively.

Finally, the feasibility and safety of valve-in-valve implants with aortic balloon expandable devices to treat stenosis or regurgitation after prior surgical TV replacement or repair has recently been reported from the voluntary unsponsored Valve-in-Valve International Database Registry.^113^ The cumulative 3-year incidence of death, reintervention, and valve-related adverse outcomes (endocarditis, thrombosis, or significant dysfunction) were 17%, 12%, and 8%, respectively. Valve-in-ring procedures pose additional issues compared to valve-in-valve procedures. Because most surgical tricuspid rings are incomplete in order to minimize the impact on the conduction system and are frequently rigid, implantation of the transcatheter valve into a rigid oval ring may result in suboptimal transcatheter heart valve leaflet function as well as a residual peri-ring leak.^114^

## Conclusion

**Figure 1:**
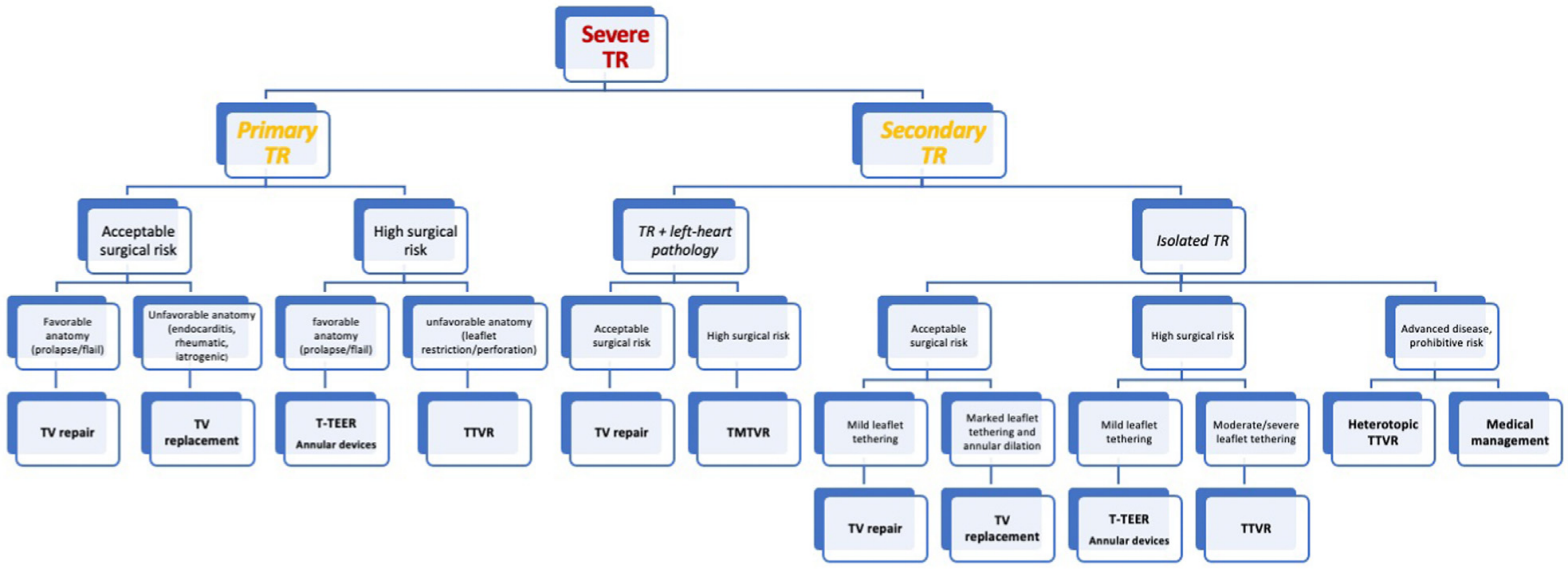
**Proposed algorithm for patient treatment**. TMTVR, transcatheter mitral and tricuspid valve repair; TR, tricuspid regurgitation; T-TEER, transcatheter tricuspid edge-to-edge repair; TTVR, transcatheter tricuspid valve replacement; TV, tricuspid valve.

Current knowledge gaps are significant. When and how to treat severe TR remains a clinical dilemma. Optimal medical treatment lacks evidence-based recommendations contributing to the undertreatment and progression of the disease. A delay in intervention until the late stages of the disease results in excessive morbidity and mortality for isolated surgical intervention. Until now, palliative therapies were frequently the only option; however, transcatheter therapies offer the hope of low-risk, efficacious treatment that is, thus far, associated with significant functional and quality-of-life improvements. Whether mortality and heart failure hospitalizations can also improve will be determined by the randomized controlled trials currently enrolling. All patients with significant symptomatic TR should be referred to a Heart Valve Center with expertise in TV disease and treatment.^16^^,^^44^ A comprehensive evaluation by the heart team is mandatory in order to determine surgical and percutaneous risks, thoroughly assess both clinical and anatomical characteristics, carefully weigh the expected results and clinical benefits versus the potential risk factors, and finally identify the most appropriate treatment strategy for every patient (Figure 1).
